# Human guanylate-binding protein (GBP) 1 inhibits replication of severe acute respiratory syndrome coronavirus 2

**DOI:** 10.1128/jvi.00823-25

**Published:** 2025-09-15

**Authors:** Rubaiyea Farrukee, Francesca Mordant, Charley Mackenzie-Kludas, Dejan Mesner, Masahiro Yamomoto, Clare Jolly, Andrew G. Brooks, Kanta Subbarao, Sarah L. Londrigan, Patrick C. Reading

**Affiliations:** 1 Department of Microbiology and Immunology, The University of Melbourne at the Peter Doherty Institute for Infection and Immunity, Melbourne, Victoria, Australia; 2 Division of Infection and Immunity, University College London 4919, London, United Kingdom; 3 Department of Immunoparasitology, Research Institute for Microbial Diseases, Osaka University, Osaka, Japan; 4 WHO Collaborating Centre for Reference and Research on Influenza, Victorian Infectious Disease Reference Laboratory, The Peter Doherty Institute for Infection and Immunity 534133 , Melbourne, Victoria, Australia; 5 Department of Microbiology and Immunology, Faculty of Medicine, Universite Laval 4440, Québec City, Canada; The Ohio State University, Columbus, Ohio, USA

**Keywords:** innate immunity, restriction factors, GBP1, SARS-CoV-2

## Abstract

Severe respiratory syndrome coronavirus 2 (SARS-CoV-2) infections are associated with significant morbidity and mortality worldwide. Identification and characterization of intracellular proteins with antiviral activity (known as restriction factors) is a key first step towards the future development of novel host-directed antiviral therapies. In this study, we investigated the antiviral activity of 14 different interferon-stimulated gene (ISG) proteins against SARS-CoV-2. Overexpression of human guanylate binding protein (GBP) 1 resulted in potent inhibition of the ancestral SARS-CoV-2 strain, as well as against Alpha, Beta, Delta, Omicron BA.1, and Omicron BA.2 variants of concern (VOCs). Moreover, knockdown or knockout of endogenous human GBP1 resulted in enhanced titers of SARS-CoV-2. While hGBP1 can restrict some viruses via actin remodeling, our data indicate that this is not the mechanism here. Moreover, we show that unlike GBP2 and GBP5, which impair spike protein processing, GBP1 did not reduce infectivity of spike-pseudotyped viruses following titration on Caco-2 cells. Multiple human GBPs have been reported to inhibit SARS-CoV-2 *in vitro*; however, we report no significant differences in virus titers recovered from the upper or lower airways of SARS-CoV-2-infected wild-type mice compared to from mice lacking the chromosome 3 cluster of mouse GBPs (GBP1/2/3/5/7). Together, our studies describe the ability of human GBP1 to inhibit SARS-CoV-2 replication, highlighting this host protein as a potential target for the development of host-directed antiviral therapies against this virus.

**IMPORTANCE:**

Viruses like SARS-CoV-2 can cause widespread illness and death. While currently licensed antiviral drugs are critical tools, drug resistance can develop. Our immune system produces intracellular proteins called “restriction factors” that can limit virus replication within cells. These proteins are promising targets for developing new antiviral therapies. In this study, we identified one such protein, human GBP1, which inhibited a range of SARS-CoV-2 variants *in vitro*, including Delta and Omicron. Interestingly, GBP1 inhibited SARS-CoV-2 through a different mechanism to that of other human GBPs as it did not interfere with prosessing of the viral spike protein. Of interest, a cluster of mouse GBPs, including GBP1, did not demontrate significant antiviral activity in a mouse model of infection. Overall, our findings suggest that human GBP1 could be a valuable target for host-directed antiviral strategies and highlight the limitations of using mouse models to study certain aspects of human innate immunity.

## INTRODUCTION

Severe respiratory syndrome coronavirus 2 (SARS-CoV-2) is a positive-sense single-stranded RNA virus from the betacoronavirus family and the causative agent of the COVID-19 pandemic (1). SARS-CoV-2 infections have been associated with over a million deaths and remain a significant global health burden (2). Vaccines and antivirals are now available for disease management; however, waning immunity and the development of antiviral resistance have also been reported (3, 4). Understanding the biology of SARS-CoV-2 is of continued importance to provide insights into the design of novel antiviral therapies to combat COVID-19-associated disease.

Recognition of viral components (pathogen-associated molecular patterns [PAMPs]) by cellular pattern recognition receptors (PRRs) activates intracellular signaling cascades, resulting in the release of type I, II, and/or III interferons (IFNs) (5, 6). These, in turn, bind to and signal through their cognate cellular receptors in both an autocrine and paracrine manner, resulting in the transcriptional activation of hundreds of interferon-stimulated genes (ISGs) that collectively induce an antiviral state, inhibiting viral replication within infected cells and rendering neighboring cells refractory to infection. Many ISG gene products and other intracellular proteins can mediate antiviral activity against particular viruses, and these collectively are referred to as restriction factors. Characterization of such proteins is of interest as they represent potential targets for the development of novel host-directed antiviral therapies (7, 8). While hundreds to thousands of ISGs can be induced within cells in response to IFNs, the antiviral activities of relatively few have been characterized in detail. Examples of well-characterized ISGs include myxovirus resistance (Mx) proteins, which inhibit replication of some RNA and DNA viruses through diverse mechanisms (reviewed in reference 9), and membrane-bound interferon-induced transmembrane (IFITM) proteins, which have been shown to inhibit entry of different viruses into host cells, particularly enveloped RNA viruses (reviewed in reference 10).

In recent years, several ISG proteins have been reported to mediate antiviral activity against SARS-CoV-2. Examples include Ly6e which inhibits viral entry (11), RTP4 which inhibits viral RNA synthesis (12), and BST-2/tetherin which prevents release of newly synthesized SARS-CoV-2 virions (13, 14). Two recent ISG-targeted CRISPR screens have also identified additional ISGs, namely OASL and DAXX, that were subsequently shown to inhibit SARS-CoV-2 replication *in vitro* (15, 16). Studies examining transcriptional responses in lung epithelial cells have reported that other ISGs, including IFI44, IFI6, IFIT1, IFIT3, and GBP1, 4, and 5, are also upregulated following SARS-CoV-2 infection (17–19). Several of these ISG proteins have been shown to modulate the replication of different viruses; however, with the exception of GBP5, IFIT1, and IFIT3, their ability to inhibit SARS-CoV-2 replication has not been reported (20, 21). A recent study reported that overexpression of GBP2 and GBP5 in virus-producing cells reduced the infectivity of pseudotyped viruses (PVs) expressing the SARS-CoV-2 spike protein and that silencing of endogenous GBP2/5 rescued IFN-γ-mediated inhibition of infectious SARS-CoV-2 isolates (20). Furthermore, both GBP2 and GBP5 were shown to inhibit furin-mediated processing of viral spike proteins, blocking protein maturation and therefore virus infectivity (20). Of the seven human GBPs, only GBP2 and GBP5 have been reported to mediate antiviral activity against SARS-CoV-2 to date.

In this study, we generated 293T-ACE2 cells with stable overexpression of IFI44, IFI6, or different members of the IFIT and GBP families and assessed the ability of these different ISG proteins to modulate SARS-CoV-2 replication *in vitro*. 293T-ACE2 cells expressing tetherin, an ISG previously reported to inhibit SARS-CoV-2 infection (13), were generated and included in experiments as a positive control for antiviral activity. We demonstrate that overexpression of GBP1 resulted in significant inhibition of productive SARS-CoV-2 replication *in vitro*. Subsequent analysis demonstrated that GBP1-mediated restriction of SARS-CoV-2 did not affect genomic replication and transcription but did inhibit the synthesis of new viral proteins. GBP1 also required functional GTPase activity to inhibit SARS-CoV-2 replication. In contrast to GBP2/5 (20), GBP1-mediated inhibition of SARS-CoV-2 did not affect viral infectivity. Moreover, while the ability of GBP2 and GBP5 to inhibit only certain SARS-CoV-2 variants of concern (VOCs) could be mapped to the viral spike protein, GBP1 restricted both ancestral and all five VOCs tested in our studies. GBP1 has been previously reported to restrict replication of Kaposi’s Sarcoma-associated herpesvirus (KSHV) through disruption of actin filaments (22). However, GBP1-mediated inhibition of SARS-CoV-2 was still observed in cells treated with a drug that disrupts F-actin rearrangement. Overall, this study is the first to report inhibition of SARS-CoV-2 by GBP1 and to demonstrate that this occurs through a mechanism distinct to that previously reported for GBP2/5.

## MATERIALS AND METHODS

### Cells and viruses

Human embryonic kidney (HEK)293T cells (ATCC CRL-3216), lung carcinoma Calu3 cells (ATCC HTB-55), Vero cells, and colorectal carcinoma Caco2 cells (ATCC HTB-38) were maintained and passaged in Dulbecco’s modified Eagle medium (DMEM10; Gibco) supplemented with 10% (vol/vol) fetal calf serum (FCS, Gibco, Thermo Fisher Scientific, MA, USA), 2 mM L-glutamine (Gibco), non-essential amino acids (Gibco), 0.55% (wt/vol) sodium bicarbonate (Gibco), 20 mM HEPES (Gibco), 200 units (U)/mL penicillin (Gibco), and 200 µg/mL streptomycin (Gibco). Lung carcinoma A549 cells (CCL-185) were maintained and passaged in Ham’s F-12K (Kaighn’s) medium (F-12K10; Gibco) supplemented with 10% (vol/vol) FCS and additional supplements as described above. Cells were maintained at 37°C and 5% CO_2._

The viruses used for these experiments were as follows: Vic01 (SARS-CoV-2/Australia/VIC01/2020; GenBank accession number: MT007544.1), Alpha (SARS-CoV-2/Australia/VIC17991/2020 [B.1.1.7]; GISAID: EPI_ISL_779606), Beta (SARS-CoV-2/Australia/ QLD1520/2020 [B.1.351]; GISAID: EPI_ISL_968081), Delta (SARS-CoV-2/Australia/VIC18440/2021 [B.1.617.2]; GISAID: EPI_ISL_1913206), Omicron BA.1 (SARS-CoV-2/Australia/ NSW/RPAH-1933/2021 [B.1.1.529.1]; GISAID: EPI_ISL_6814922), and Omicron BA.2 (SARS-CoV-2/Australia/VIC35864/2022 [B.1.1.529.2]; GISAID: EPI_ISL_8955536). All viruses were kindly provided by the Victorian Infectious Diseases Reference Laboratory (Melbourne, Australia).

Vic01 and the Alpha, Beta, and Delta VOCs were propagated in Vero cells kindly provided by Dr. Julian Druce (Victorian Infectious Diseases Reference Laboratory, Australia), and maintained in Minimum Essential Media (MEM) supplemented with 100 U/mL penicillin, 100 µg/mL streptomycin, 2 mM GlutaMAX (Gibco), 15 mM HEPES, 400 µg/mL Geneticin (Gibco), and 1 µg/mL TPCK trypsin (Worthington), for 4 days before harvesting viral supernatant and storing in aliquots at −80°C. Omicron BA.1 and BA.2 viruses were propagated in Calu3 cells cultured in MEM supplemented with 100 U/mL penicillin, 100 µg/mL streptomycin, 1% non-essential amino acids, 1 mM sodium pyruvate, and 2% FCS for 4 days, before supernatants were harvested, clarified, and stored at −80°C.

All SARS-CoV-2 viruses were titrated on Vero cells. Briefly, serial 10-fold dilutions of virus were performed on monolayers of Vero cells in DMEM supplemented with 100 U/mL penicillin, 100 µg/mL streptomycin, 2 mM GlutaMAX, and 1 µg/mL TPCK trypsin. Plates were incubated at 37°C with 5% CO_2_ for 4 days before reading cytopathic effect and calculating viral titer using the Reed–Muench formula (23).

### Generation of cell lines with stable constitutive expression of ACE2 and ISG proteins

293T and A549 cells with stable expression of human ACE2 were generated using retroviral transduction. Briefly, 293T cells seeded in 10 cm^2^ dishes were cultured overnight and then transfected using Fugene HD transfection reagent (Promega #E2311) with 4 ug PMIGII.ACE2 (which co-expresses GFP, Addgene #52107), 4 µg pPam-E (pEQ-Pam3(-E)) plasmid, and 2 µg pVSVG (24) kindly provided by Dr. Nicholas Gherardin (Department of Microbiology and Immunology, University of Melbourne). At 24 and 48 h post-transfection, supernatant from transfected 293T cells was filtered through 0.45 µM filter and used to transduce target cells in the presence of 8 µg/mL polybrene (Sigma, #H9268). After transduction and expansion, target cells were sorted twice using a FACS ARIA III to select for GFP^+^ cells. Cell surface expression of ACE2 was confirmed on transduced cells using an ACE2-specific mAb (Invitrogen, #MA532307) in conjunction with flow cytometric analysis.

To generate cell lines with stable overexpression of particular ISGs, each ISG, with an N-terminal FLAG tag, was cloned into pcDNA3.1 expression plasmids (Invitrogen) modified to express the IRES mCherry element as described (25). pcDNA3.1 expressing cytoplasmic ovalbumin and lacking a FLAG tag was also generated as a control (CTRL) (26). After cloning, inserts were confirmed by sequencing, and 1 µg of each plasmid was transfected into 293T-ACE2 cells using Lipofectamine 2000 (Life Technologies, CA, USA). Cells were cultured at 37°C for 2 days before the media was supplemented with 100 µg/mL hygromycin B (InvivoGen, #10687-010). After culture for >10 days, mCherry^+^ cells were sorted twice to enrich for stably transfected cells for use in subsequent experiments. To generate GBP1-K51A cells, the K51A mutation was introduced in the GBP1 pcDNA3.1-mCherry vector by site-directed mutagenesis using the GeneART Site-Directed Mutagenesis Kit (Invitrogen), and cells with stable overexpression of FLAG-tagged GBP1-K51A were generated as described. Sequences of human ACE2 and all ISGs studied are available in Table S1. Flow cytometry was then used to detect intracellular expression of FLAG-tagged proteins in 293T-ACE2 cells. Cells were stained with fixable viability dye eFluor 450 (eBioscience), then fixed, permeabilized, and stained with allophycocyanin (APC)-conjugated anti-FLAG mAb (clone L5; Biolegend, #637307) prior to analysis by flow cytometry using a BD LSR Fortessa (BD Biosciences).

### Virus infection experiments

293T-ACE2 cells were seeded in poly-L-lysine–treated 24-well plates (Thermo Fisher, #A3890401) at 2.5 × 10^5^ cells/well and cultured for 24 h prior to infection. Cells were washed once in serum-free media and then incubated with virus inoculum for 2 h at 37°C. Virus inoculum was then removed, cells were washed once, and then incubated at 37°C in serum-free media containing 0.5 ug/mL TPCK trypsin (Worthington, #LS003740). At the indicated time points, cell-free supernatants were harvested, and virus titers were determined by TCID_50_ assay using Vero cells as described previously (23) or by qRT-PCR.

For qRT-PCR, viral RNA was extracted using QIAamp Viral RNA Extraction Kit (Qiagen, #52906). The amount of viral E gene in 5 uL of extracted RNA was then quantified using the SensiFAST Probe No-ROX One-Step Kit (Bioline, #76005), using the following primers and probes: Forward: 5′ ACAGGTACGTTAATAGTTAATAGCGT 3′, Reverse: 5′ ATATTGCAGCAGTACGCACACA 3′, and Probe: 5′ FAM- ACACTAGCCATCCTTACTGCGCTTCG-TAMRA 3′. Extracted viral RNA was then analyzed alongside an E gene plasmid standard curve (kindly provided by Dr. Brad Gilbertson, Department of Microbiology and Immunology, University of Melbourne) to quantify copy numbers of viral RNA per mL of original sample. Data acquisition was performed using the QuantStudio 7 Pro Real-Time PCR System (Applied Biosystems).

### Detection of SARS-CoV-2 genomic replication and transcription in infected cells

293T-ACE2 cells stably expressing proteins of interest were seeded and infected with SARS-CoV-2 as described above or mock infected with serum-free media. At 24 h post-infection (hpi), total cell lysates were collected in TRIzol reagent (Invitrogen, #15596026) and RNA extracted using phenol-chloroform extraction, as per manufacturer’s instructions. DNA was removed using amplification grade DNase I (Sigma-Aldrich, #AMPD1), and then the RNA concentration was standardized across samples. One set of RNA was then transcribed into cDNA using the SensiFast cDNA Synthesis Kit (Bioline, #65053), and SYBR green-based qPCR was used to analyze the expression of E gene subgenomic RNA as well as two housekeeping genes: GAPDH (glyceraldehyde 3-phosphate dehydrogenase) and TBP (TATA-binding protein) (26) using the SensiFAST SYBR Lo-ROX Kit (Bioline, #94020). Data acquisition was performed using the QuantStudio 7 Pro Real-Time PCR System (Applied Biosystems). Primers used to detect subgenomic E are as follows: Forward: 5′ CGATCTCTTGTAGATCTGTTCTC 3′ and Reverse: 5′ ATATTGCAGCAGTACGCACACA 3′. Fold change of subgenomic E gene from mock was calculated using 2^-ΔΔCT^ methods (27).

To detect poly A-tailed E gene RNA, enrichment was performed during cDNA synthesis, using Oligo dT primers and the Tetro cDNA synthesis kit (Bioline, #65042). The level of E gene was then determined relative to mock and normalized to housekeeping genes by qPCR as above but using the following E gene-specific primers: Forward: 5′ ACAGGTACGTTAATAGTTAATAGCGT 3′, Reverse: 5′ ATATTGCAGCAGTACGCACACA 3′.

### Detection of viral proteins in SARS-CoV-2-infected cells

293T-ACE2 cells stably overexpressing proteins of interest were seeded and infected with SARS-CoV-2 as described above or mock infected with serum-free media. At the indicated times post-infection, cells were fixed in 4% (vol/vol) paraformaldehyde (ProSciTech, #C004), permeabilized, and stained for either intracellular expression of viral (a) NP (CR3009, Abcam, #ab275984) or (b) ORF3a (Abcam #ab280953), followed by anti-rabbit secondary Alexa Fluor 488 (Invitrogen, #11048) as previously described (25). Cells were then analyzed by flow cytometry. For western blot, total cell lysates were collected in protein lysis buffer, and proteins were separated by SDS-PAGE (15% vol/vol) prior to transfer to polyvinylidene difluoride membranes (PVDF) (Immobilon-P, Merck Millipore) as previously described (25, 26). Membranes were blocked with blocking buffer containing 5% bovine serum albumin (Sigma-Aldrich) in PBS with 0.1% Tween-20 (Sigma-Aldrich), prior to incubation with (i) 0.1 µg/mL rabbit anti-spike mAb (Abcam, #272504) and (ii) 1 µg/mL mouse anti-actin mAb as loading control (Santa Cruz Biolabs, SC4778) at 4°C overnight in blocking buffer. After washing with PBS containing 0.1% Tween-20, membranes were incubated with appropriate secondary antibodies (Alexa Fluor 647 anti-rabbit IgG or Alexa Fluor 488 anti-mouse IgG [Invitrogen]) for 1 h at room temperature, and after washing, images were acquired using an Amersham Imager 800 (GE Healthcare). Relative intensity to loading controls was calculated using ImageJ (28).

### Knockdown or knockout of endogenous GBP1

To detect expression of endogenous GBP1, monolayers of 293T-ACE2, A549-ACE2, and Calu3 cells were incubated with different concentrations of recombinant mouse IFN-γ (Biolegend, #570202) or media alone (mock) for 24 h at 37°C. After incubation, RNA was extracted using the RNeasy Plus Mini Kit according to manufacturer’s instructions (Qiagen) and treated with amplification grade DNase I (Sigma-Aldrich), and then, RNA concentrations were standardized across samples. RNA was transcribed into cDNA using the SensiFast cDNA Synthesis Kit (Bioline), and SYBR green-based qPCR was used to analyze the expression of GBP1 relative to housekeeping genes GAPDH and TBP using the SensiFAST SYBR Lo-ROX Kit. Data acquisition was performed using the QuantStudio 7 Pro Real-Time PCR System (Applied Biosystems). Primers used for GBP1 were as follows: Forward: 5′ GGTCCAGTTGCTGAAAGAGC 3′ and Reverse: 5′ TGACAGGAAGGCTCTGGTCT 3′.

For siRNA-mediated knockdown (KD), A549-ACE2 or Calu3 cells were seeded into 24-well tissue culture plates in Opti-MEM Reduced Serum Medium (Gibco) supplemented with 5% (vol/vol) FCS and incubated overnight. Cells were then transfected with 10 nM of GBP1-specific siRNA or with non-targeting control (NTC) (SiGENOME SMART pool, Dharmacon, CO, USA) using Lipofectamine RNAiMAX (Thermo Fisher Scientific), according to manufacturer’s instructions, and incubated at 37°C. After 24 h, the cells were then treated with either 100 ng/uL IFN-γ (A549-ACE2 cells) or 50 ng/uL IFN-γ (Calu3 cells) and incubated for an additional 24 h, and then, either (i) lysates were generated for subsequent qPCR analysis or (ii) cells infected with SARS-CoV-2 (Vic01, multiplicity of infection [MOI] 1) and incubated at 37°C. At 24 hpi, the percentage of virus-infected cells was determined using flow cytometry to detect cells expressing the viral NP protein as described above.

For CRISPR/Cas9 editing, specific guide RNA (sgRNA) for GBP1 or scrambled guides were obtained from Synthego. The sgRNA and Cas9 protein (Alt-R S.p. Cas9 Nuclease V3, IDT, #1081059) were then used for genome editing using the Lonza 4D Nucleofection System (Lonza), according to manufacturer’s instructions. Cells were transfected using the nucleofector machine (4D-Nucleofector X, Lonza) with pre-programmed settings (CM-1-03) suitable for A549 cells. After nucleofection, cells were seeded into 12-well tissue culture plates and expanded for subsequent experiments. Expanded cells were subject to a second round of nucleofection with Cas9 and sgRNA prior to experiments. To detect endogenous GBP1, cells were pre-treated with 100 ng/µL of IFN-γ, and cell lysates generated 24 h later were analyzed using western blotting as described above. Expression of endogenous GBP1 was detected by incubation with rabbit anti-GBP1 mAb ([EPR8285, #ab131255), followed by anti-rabbit Alexa Fluor 647 antibody (Invitrogen, #A-31573). For SARS-CoV-2 infection, GBP1 knockout (KO) or scrambled sgRNA-treated cells cultured overnight in 24-well plates were treated with 100 ng/µL of IFN-γ for 24 h, infected with SARS-CoV-2 (Vic01 MOI 1), and incubated at 37°C. At 24 hpi, the percentage of virus-infected cells was determined using flow cytometry to detect cells expressing the viral NP protein as described above.

### Generation of SARS-CoV-2 spike-expressing pseudotyped viruses

Pseudotyped virus experiments were performed as previously described (20). Briefly, spike-pseudotyped viruses (PVs) were made by co-transfection of spike (40 ng), p8.91 (260 ng), and pCSLW (260 ng) vectors, alongside 120 ng of GBP-expressing or control vectors, in 293T/17 cells (ATCC CRL-11268) seeded in 24-well plates. PV supernatants were collected at 48 and 72 h post-transfection, purified through 0.45 µm centrifuge tube filters (Corning), and used within 24 h without freeze–thawing. The amount of PV in the supernatant was determined by measuring reverse transcriptase (RT) activity using SYBR-green-based product-enhanced reverse transcription assay (SG-PERT) by qPCR, as described previously (29). For PV titration, Caco2 cells seeded into white 96-well plates were cultured for 24 h and then infected with 2-fold dilutions of PV supernatant (to confirm linear decrease in infection with dilution). After incubation at 37°C for 72 h (without changing media), luciferase expression (relative light units, RLU) was measured using BrightGlo substrate (Promega) according to manufacturer’s instructions, using a Synergy H1 luminometer (BioTek). Infectivity of the supernatant was then determined as a ratio of RLU to RT units (RLU/RT).

### Assessment of actin in modulating GBP1-mediated inhibition of SARS-CoV-2

To determine the impact of endogenous GBP1 on actin filament structure, A549-ACE2 scrambled and GBP1 KO cells were seeded in coverslips, cultured overnight, and then treated with 100 ng/µL of IFN-γ for 24 h. Cells were then fixed with 4% paraformaldehyde (ProSciTech, #C004), permeabilized with 0.1% (vol/vol) Triton X-100 (Sigma Aldrich), and stained with Alexa Fluor PLUS 647 Phalloidin (Thermo Fisher, #A30107) and 4’,6-diamidino-2-phenylindole (DAPI), and then mounted using ProLong Gold (Life Technologies) mounting media. To detect if overexpression of exogenous GBP1 affected actin filament structure, 8-well ibidi chamber slides (DKSH, #80826-90) were pre-treated with 0.01% poly-L-lysine (Sigma, #P8920) and 0.01% fibronectin (Sigma, #F0895), seeded with 293T-ACE2-CTRL and -GBP1 cells, cultured overnight, and then stained for actin as described above. All images were acquired using Zeiss LSM 980 laser scanning microscopy. To test if the actin pathway is involved in GBP1-mediated restriction of SARS-CoV-2, 293T-ACE2-CTRL and -GBP1 cells were infected with SARS-CoV-2 (Vic01) at MOI 0.5 and then, 8 h later, treated with either 20 µM of K-252A (Santa Cruz, #200517) or an equivalent volume of DMSO (carrier). At 24 h post-infection, cells were fixed and stained for intracellular expression of viral NP staining and analyzed by flow cytometry.

### Mouse experiments

Heterozygote GBPchr3 KO mice (from Prof. Masahiro Yamamoto, Department of Immunoparasitology, Research Institute for Microbial Diseases [RIMD], Osaka University, Japan [30]) was bred to generate homozygous wild-type (WT) and GBPchr3 KO mice to ensure consistent genetic background. Mice were bred and housed in specific pathogen-free conditions in the Bioresources Facility of the Peter Doherty Institute for Infection and Immunity, Melbourne, Australia. All research complied with the University of Melbourne’s Animal Experimentation Ethics guidelines and policies, and all experimental protocols were approved by the University of Melbourne Animal Ethics Committee (Approval numbers ^#^1814689 and ^#^20763). Mice were kept in hermetically sealed, individually vented cages at the AgriBio Center for AgriBioscience BSL3 animal facility.

For SARS-CoV-2 infections, WT or GBPchr3 KO mice (6–8 weeks of age) were anesthetized with 3% isoflurane (v0l/vol with 2 L/min oxygen) and transduced via the intranasal route with 2.5 × 10^8^ focus-forming units (FFU) of Ad5-ACE2, a replication-incompetent adenovirus expressing hACE2 (purchased from Water Eliza Health Institute Bioresource Facility, Victoria, Australia [Cat no. #NR 52390]), in 75 µL of PBS (31, 32). At day 5 post-transduction, mice were anesthetized and infected via the intranasal route with 10^4^ TCID_50_ of SARS-CoV-2 strain Vic01 in 50 µL PBS. Mice were monitored for clinical signs of disease and weighed daily for the duration of each experiment. Infected mice were monitored for weight loss signs until 14 days post-infection, or mice were euthanized at day 2 and/or 4 post-infection, and lungs and nasal turbinates were collected, homogenized in PBS, and clarified by centrifugation. Titers of infectious SARS-CoV-2 in clarified homogenates were determined by TCID_50_ assay.

### Statistical analysis

Graphs and statistical analysis (as indicated in the figure legends) were performed using R software version 4.0.3. Flow cytometry data were analyzed using FlowJo version 10 (Becton, Dickinson and Company). Image analysis was performed using Fiji (33).

For pooled data in Fig. S3a, mixed effects model (*nlme* package in R) was used to estimate the fixed effects (e.g., difference between CTRL and GBP2 cells) while controlling for similarities between technical replicates (random effects) in each independent experiment (biological replicates).

## RESULTS

### Overexpression of different ISGs identifies GBP1 as a novel restriction factor for SARS-CoV-2

**Fig 1:**
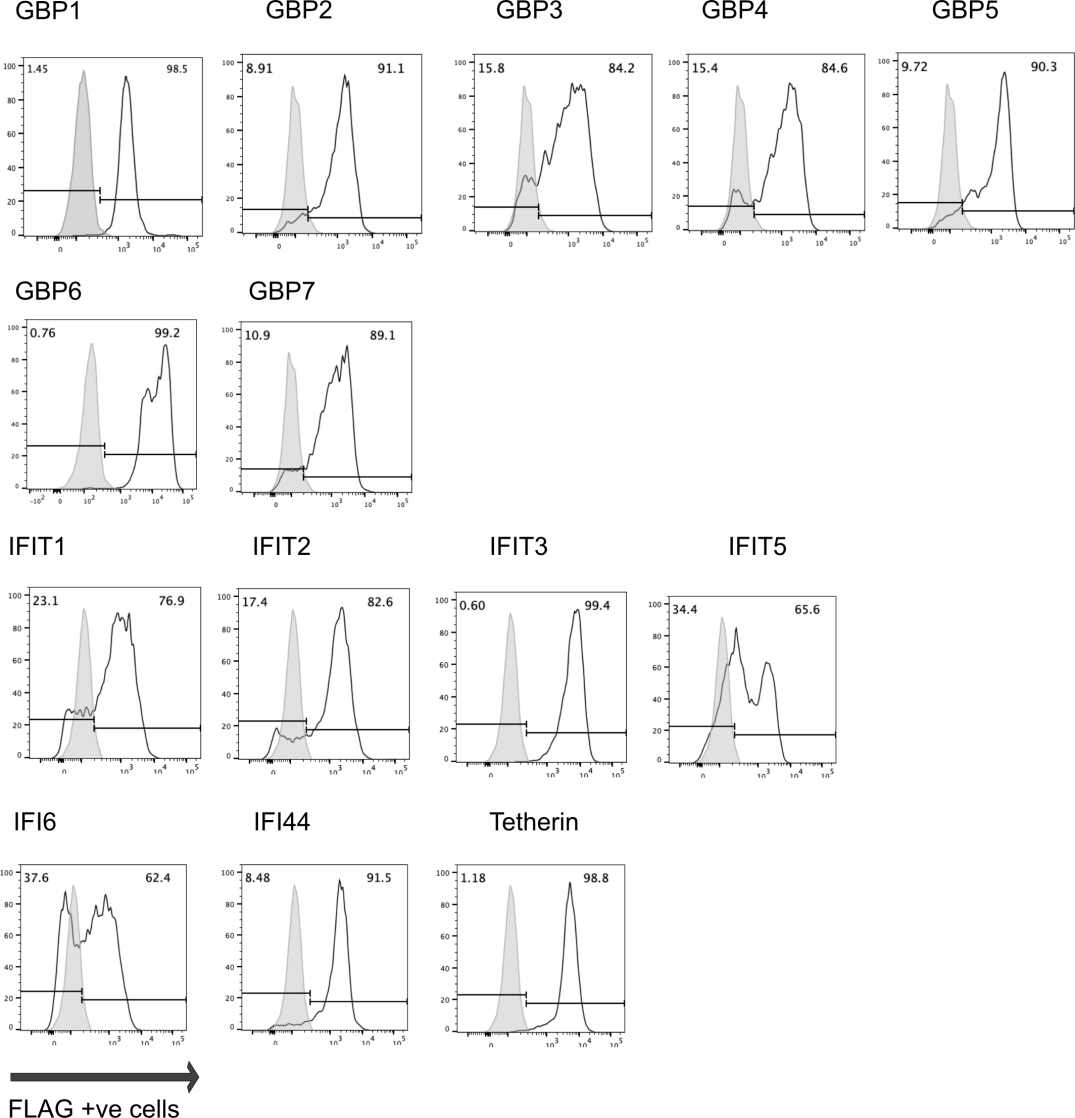
Generation of 293T-ACE2 cells with stable overexpression of different ISG proteins. 293T-ACE2 cells were transfected with pcDNA3.1-mCherry vectors expressing ISG proteins of interest, each with a N-terminal FLAG tag, or with the same vector expressing cytoplasmic chicken ovalbumin with no FLAG tag as a control (CTRL). Stable transfectants were selected in the presence of hygromycin and enriched by sorting for mCherry^+^ cells. Cells were fixed and stained for intracellular expression of FLAG-tagged proteins and examined by flow cytometry. After gating on live mCherry^+^ cells, FLAG expression was determined in different ISG-expressing cell lines (white histograms) relative to CTRL cells (gray histogram).

**Fig 2:**
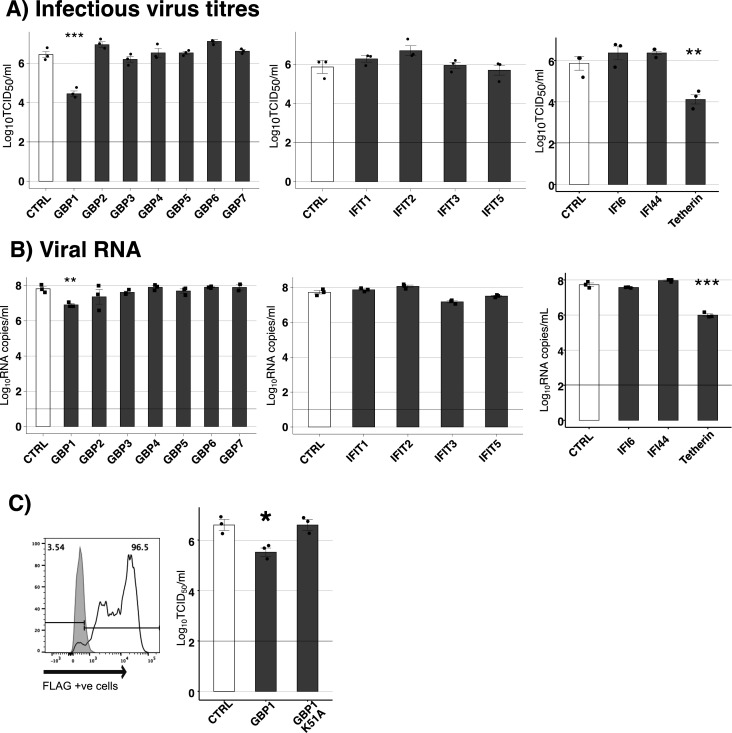
Cell lines with stable overexpression of GBP1 or tetherin inhibit growth of SARS-CoV-2. 293T-ACE2 CTRL cells or cells overexpressing FLAG-tagged ISG proteins were infected with SARS-CoV-2 (Vic01, MOI 0.1) for 1 h at 37°C, washed, and then incubated at 37°C. At 48 hpi, supernatants were collected, clarified, and used to determine (A) titres of infectious virus by TCID_50_ assay on Vero cells, or (B) viral RNA copy number using primers specific to the viral E gene. Triplicate samples are shown, noting that the inhibition of SARS-CoV-2 by both GBP1 and tetherin was confirmed in independent experiments. (C) 293T-ACE2 cells stably overexpressing a catalytically inactive FLAG-tagged GBP1 (GBP1-K51A) were generated and expression of FLAG-tagged protein confirmed by flow cytometry. 293T-ACE2 cells overexpressing either CTRL, GBP1, or GBP1-K51A were then infected with SARS-CoV-2 (Vic01, MOI 0.1), washed, and incubated for 48 h before virus titers in clarified supernatants were determined by TCID_50_ assay on Vero cells. Data show triplicate samples from one of two independent experiments performed with similar results. The limit of detection for TCID_50_ and qPCR is shown by horizontal lines. Statistical analysis was performed using Student’s unpaired *t*-test with unequal variance to compare cell lines expressing ISG to the CTRL cell line. *P < 0.05, **P < 0.01, ***P < 0.001.

To test if different restriction factors could inhibit SARS-CoV-2 replication, we first generated human epithelial cell lines (293T and A549) overexpressing human ACE2 receptors to increase their susceptibility to support productive replication of SARS-CoV-2 (Fig. S1A and C). 293T-ACE2 cells were then transfected with pcDNA3.1-mCherry vectors encoding human GBP1-7, IFIT1-5, IF44, IFI6, or tetherin, each with a N-terminal FLAG tag. Cells were enriched for stable transfectants by culturing in the presence of hygromycin and cell sorting for mCherry^+^ cells. A control cell line (CTRL) was generated in a similar manner following transfection with pcDNA3.1-mCherry encoding an irrelevant intracellular protein, cytoplasmic chicken ovalbumin, which did not impact growth of SARS-CoV-2 (Fig. S1D). Staining of stable transfectants for intracellular FLAG-tagged proteins confirmed high (>80%) levels of FLAG-tagged ISG proteins in most cell lines, although levels of FLAG^+^ IFIT1, IFIT5, and IFI6 cell lines ranged from 60% to 70% (Fig. 1). Of note, the expression of mCherry across all cell lines was similar (>90%), except GBP6 (76%) (Fig. S2). Next, stable 293T-ACE2 transfectants were infected with SARS-CoV-2, and virus titers were determined at 48 h post-infection (hpi) by measuring (i) titers of infectious virus by TCID_50_ (Fig. 2A), or (ii) levels of viral RNA in supernatants by qRT-PCR (Fig. 2B). When compared to 293T-ACE2 CTRL cells, the only ISGs that significantly reduced viral titers were GBP1 and tetherin (>99% by TCID_50_ and >90% by qRT-PCR for both ISGs). We confirmed that the percentage of ACE2^+^ cells in GBP1- and tetherin-overexpressing 293T was similar to CTRL cells, although the peak fluorescent intensity of ACE2 in 293T-ACE2 tetherin cells was slightly lower (Fig. S1B). While GBP2 and GBP5 have been reported to restrict SARS-CoV-2 by inhibiting furin-mediated cleavage of the viral spike protein, this was not observed in our experimental system as we determined viral titers on Vero cells, a cell line in which SARS-CoV-2 entry occurs independently of furin-mediated cleavage (20, 34). While inhibition of SARS-CoV-2 by tetherin has been reported (13, 14), to our knowledge, these studies provide the first evidence that GBP1 can also restrict productive replication of SARS-CoV-2.

K51 is a conserved residue in the phosphate-binding loop of GBP1 that is crucial for its GTPase activity. The K51A mutation has been reported to abrogate the antiviral activity of GBP1 against IAV (35). Therefore, we generated 293T-ACE2 cells with stable overexpression of K51A GBP1 and confirmed intracellular expression of the FLAG-tagged protein (Fig. 2C, left panel). Moreover, the introduction of the K51A mutation into GBP1 abrogated its ability to inhibit SARS-CoV-2 replication (Fig. 2C**,** right panel), confirming the importance of intact GTPase activity for GBP1-mediated inhibition of SARS-CoV-2.

### GBP1 inhibits replication of multiple SARS-CoV-2 VOCs

**Fig 3:**
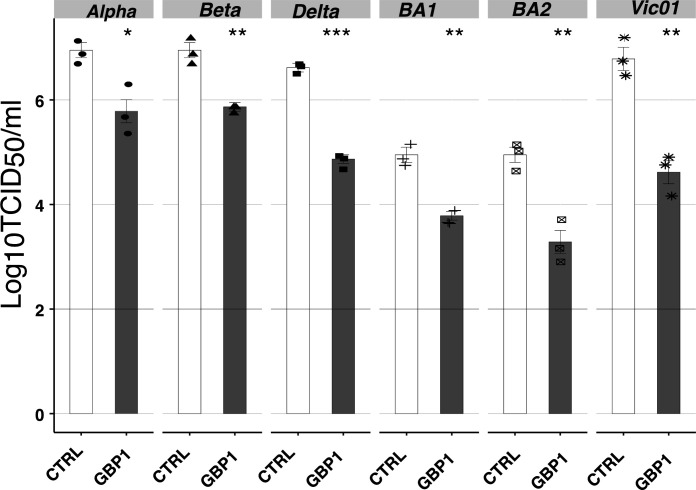
GBP1 inhibits replication of multiple SARS-CoV-2 VOCs. 293T-ACE2 CTRL or GBP1 cells were infected with different SARS-CoV-2 variants (MOI 0.1) and incubated for 48 h before virus titers in clarified supernatants were determined by TCID_50_ assay on Vero cells. Data show triplicate samples from one of two independent experiments performed with similar results. Different symbols were used for each VOC. Limit of detection for TCID_50_ is shown by horizontal lines. Statistical analysis was performed using Student’s unpaired *t*-test with unequal variance to compare cell lines expressing GBP1 to the CTRL cell line. *P < 0.05, **P < 0.01, ***P < 0.001.

A recent study reported that GBP2/5 showed differences in their ability to inhibit different SARS-CoV-2 VOCs, and this could be mapped to the viral spike protein (20). Specifically, replication-competent Vic and Omicron isolates, but not Alpha and Delta VOCs, were sensitive to inhibition by endogenous GBP2/5. To assess the impact of GBP1 on the ability of different SARS-CoV-2 VOCs to replicate *in vitro*, 293T-ACE2 GBP1 and CTRL cells were infected with Vic01 or with Alpha, Beta, Delta, Omicron BA.1, or Omicron BA.2 VOCs, and virus titers in clarified supernatants were determined on Vero cells at 48 hpi. Consistent with previous reports (36), VOCs showed differences in their ability to replicate in ACE2-A549-CTRL cells; however, all showed significant (>90%) reductions in virus titers recovered from 293T-ACE2 GBP1 cells (Fig. 3).

### GBP1 reduces SARS-CoV-2 protein levels

**Fig 4:**
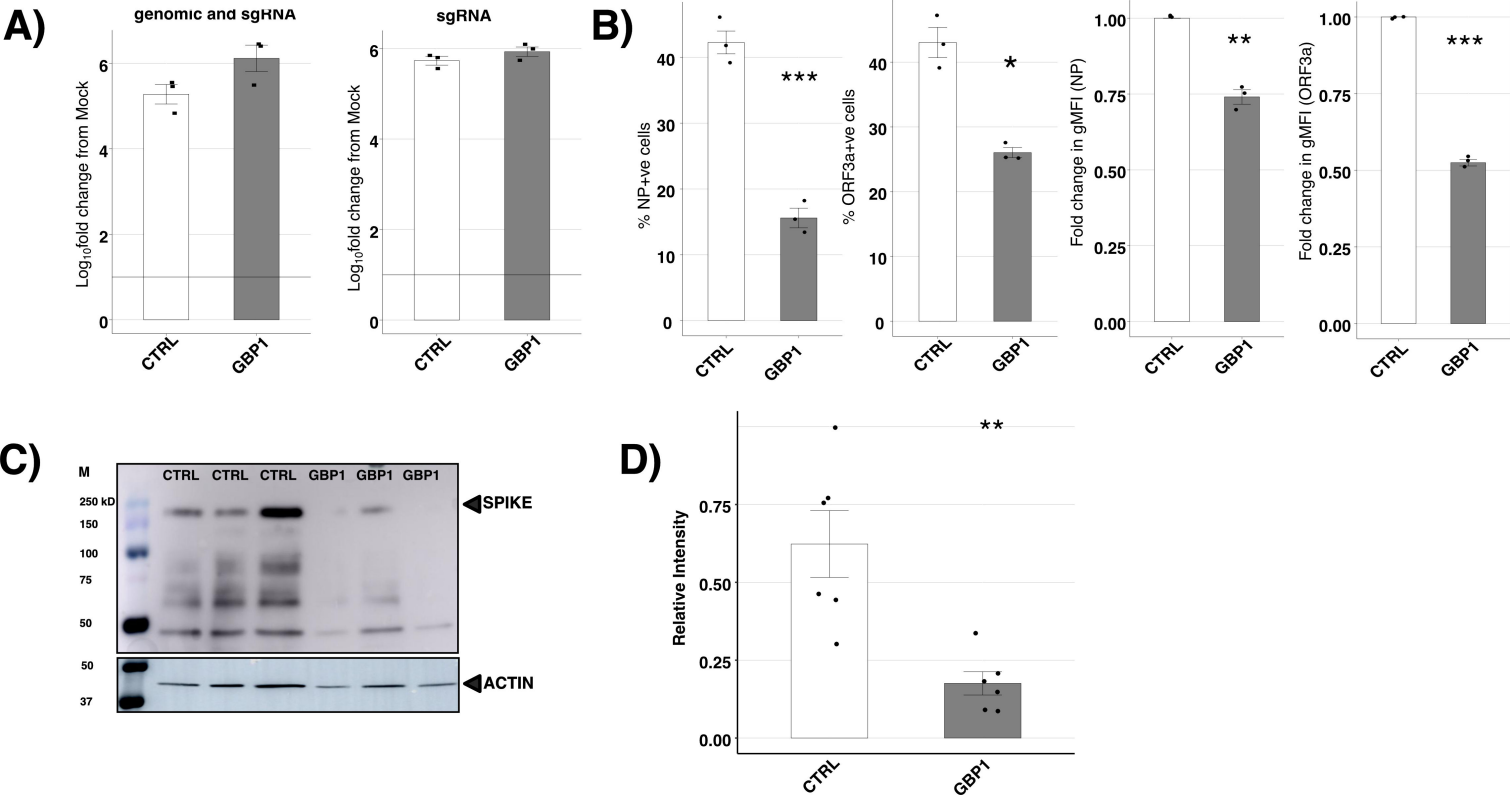
GBP1 blocks SARS-CoV-2 replication at a step following genomic transcription and replication, but prior to synthesis of new viral proteins. 293T-ACE2 CTRL or 293T-ACE2 GBP1 cells were mock-infected or infected with SARS-CoV-2 (Vic01, MOI 0.1) for 1 h, washed, and then cultured at 37°C. (A) At 24 hpi, total RNA was collected, and polyA-tailed RNA was enriched during cDNA synthesis before E gene primers were used to determine levels of SARS-CoV-2 genomic and subgenomic (sg)RNA by qPCR. Alternatively, all RNA was converted to cDNA using random hexamers, and primers specific to subgenomic E gene were used to determine only subgenomic RNA levels by qPCR. Data are shown as fold change relative to mock-infected cells after standardizing to housekeeping genes (GAPDH and TBP) and are shown on a Log_10_ scale. Triplicate samples from one of two independent experiments are shown. (B) At 24 hpi, cells were fixed, stained for intracellular expression of viral NP or ORF3a, and analyzed by flow cytometry. The percentage of NP^+^ and ORF3a^+^ cells is shown, as well as the fold change in gMFI of each viral protein relative to CTRL cells. Triplicate samples from one of three independent experiments are shown. (C) At 24 hpi, total cell lysates were collected, run on SDS-PAGE, and western blot performed for detection of viral spike protein or actin (loading control). A representative blot showing samples from three of the six independent infection experiments is shown. (D) Relative quantitation of spike protein intensity from six independent experiments as described in C. Statistical analysis was performed using Student’s unpaired *t*-test with unequal variance or Wilcoxon rank sum test (for fold change values) to compare cell lines expressing ISG to the CTRL cell line. *P < 0.05, **P < 0.01, ***P < 0.001.

To gain insight into which step in the SARS-CoV-2 replication cycle was inhibited by GBP1, we determined levels of different viral RNA species and viral proteins produced 24 h after infection of 293T-ACE2 GBP1 or CTRL cells. To assess the impact on viral RNA species, we first enriched for poly-A-tailed RNA during cDNA synthesis and used E gene-specific primers to quantitate the levels of poly A-tailed E gene RNA produced. This measurement quantifies both newly synthesized genomic RNA and subgenomic (sg) RNAs, allowing us to determine if genome replication and transcription were affected by GBP1 overexpression. Preliminary experiments in CTRL cells confirmed that the contribution of the inoculum (0.1 TCID_50_/cell) to the overall genome copy number detected at 24 hpi was minimal (Fig. S1D). As a second approach, we performed qPCR using primers specifically targeting E gene sgRNA to determine whether transcription of sgRNA was affected by GBP1 overexpression. Using either approach, we did not detect significant differences in poly A-tailed E gene RNA (genomic + sgRNA) (Fig. 4A, left panel) or sgRNA (Fig. 4A, right panel) between 293T-ACE2 GBP1 and CTRL cells.

Next, we quantified levels of SARS-CoV-2 viral proteins at 24 hpi using flow cytometry or western blot. Following staining for expression of intracellular viral nucleoprotein (NP), we observed a significant reduction in the percentage of NP^+^ 293T-ACE2 GBP1 cells and in the mean fluorescence intensity (gMFI) compared to CTRL cells (63% and 25% reductions in NP^+^ cells and in gMFI, respectively, Fig. 4B; Fig. S1). Similarly, when assessing viral ORF3a expression in SARS-CoV-2-infected 293T-ACE2-GBP1 cells, we observed 40% and 47% reductions in ORF3a^+^ cells and in gMFI relative to CTRL cells, respectively (Fig. 3; Fig. S1). By western blot, levels of spike protein detected in 293T-ACE2 GBP1 cells were also significantly reduced compared to CTRL (Fig. 4C). Together, these data demonstrate that following overexpression of GBP1, levels of SARS-CoV-2 NP, ORF3a, and spike proteins were significantly reduced even though GBP1 did not inhibit production of viral RNA transcripts in SARS-CoV-2-infected cells. Thus, GBP1-mediated inhibition of SARS-CoV-2 replication correlated with reduction of viral protein levels but had no major impacts on levels of viral genome replication or transcription.

### Endogenous GBP1 inhibits SARS-CoV-2 replication *in vitro*

**Fig 5:**
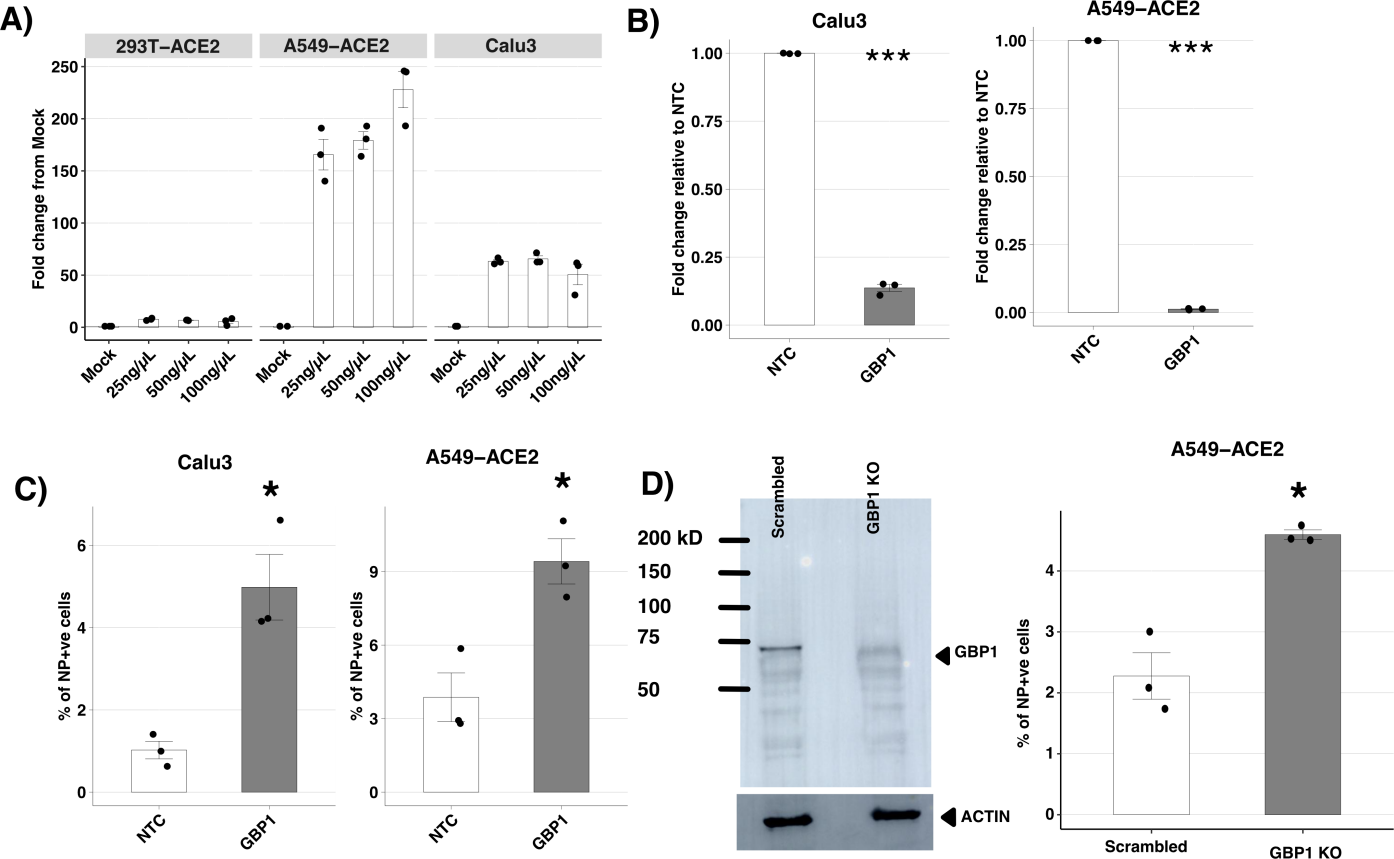
Endogenous GBP1 inhibits SARS-CoV-2 replication *in vitro*. (A) 293T-ACE2, A549-ACE2, and Calu3 cells were treated with increasing concentrations of IFN-γ for 24 h before levels of GBP1 mRNA were determined by qPCR. Induction of GBP1 is presented as fold change from mock after standardizing to housekeeping genes (GAPDH and TBP). Triplicate samples from one of two independent experiments are shown. (B and C) Calu3 or A549-ACE2 cells were treated with 10 uM of siRNA to GBP1 or with non-targeting control (NTC) siRNA for 24 h and then stimulated with either 50 ng/µL (Calu3) or 100 ng/µL (A549-ACE2) IFN-γ for another 24 h. (B) Levels of GBP1 mRNA were then determined by qPCR as for A. Triplicate samples from one of two independent experiments are shown. (C) Cells were then infected with SARS-CoV-2 (Vic01, MOI 1) for 1 h, washed, and cultured at 37°C. At 24 hpi, the % of NP^+^ cells was determined by intracellular staining and subsequent flow cytometry. Representative data from one of two independent experiments, each performed in triplicate, are shown. (D) CRISPR/Cas9 was used to knockout endogenous GBP1 (GBP1 KO) in A549-ACE2 cells. A control cell line was also generated using scrambled guide RNA (scrambled). Loss of endogenous GBP1 from GBP1 KO cells was confirmed by treating cells with 100 ng/µL of IFN-γ and, 24 h later, performing western blot using a GBP1-specific mAb with actin as a loading control (left panel). GBP1 KO or scrambled A549-ACE2 cells were also treated with 100 ng/µL of IFN-γ for 24 h and then infected with SARS-CoV-2 (Vic01, MOI 1) for 1 h, washed, and cultured at 37°C. At 24 hpi, cells were fixed, permeabilized, and stained, and the % of NP^+^ cells determined by flow cytometry. Representative data from one of two independent experiments. Statistical analysis was performed using the Wilcoxon rank sum test for fold change analysis. Student’s unpaired *t*-test was used to compare cell lines to appropriate controls. *P < 0.5, **P < 0.01, ***P < 0.001.

GBPs are ISG proteins that are most strongly induced by IFN-γ (37). Therefore, we assessed induction of endogenous GBP1 following treatment of three human cell lines susceptible to SARS-CoV-2 infection (293T-ACE2, A549-ACE2, and Calu3 cells), with increasing concentrations of recombinant human IFN-γ (Fig. 5A). While 293T-ACE2 cells showed low levels of GBP1 induction in response to IFN-γ, GBP1 was strongly upregulated in both Calu3 cells and A549-ACE2 cells (40- to 50-fold in Calu3 vs. 200-fold increase in A549-ACE2 cells following treatment with 50–100 ng/µL IFN-γ).

Next, we demonstrated efficient siRNA-mediated knockdown of GBP1 in IFN-γ-treated Calu3 and A549-ACE2 (>85% and >95% reduction in GBP1 mRNA levels, respectively) (Fig. 5B). Moreover, siRNA-mediated knockdown of endogenous GBP1 in IFN-γ-treated cells resulted in a significant increase in the percentage of NP^+^ cells in Calu3 and A549-ACE2 cells relative to NTC-treated control cells (Fig. 5C). We also utilized CRISPR/Cas9 to knockout (KO) endogenous GBP1 expression in A549-ACE2 cells. After western blot confirmed loss of GBP1 protein expression in GBP1 KO cells (Fig. 5D, left panels), we pre-treated cells with 100 ng/µL of IFN-γ, then infected with SARS-CoV-2, and used flow cytometry to detect NP^+^ cells at 24 hpi. Knockout of endogenous GBP1 resulted in a significant increase in the percentage of NP^+^ cells when compared to control cells treated with scrambled guide RNA (Fig. 5D, right panels). These results indicate that endogenous GBP1 can mediate antiviral activity against SARS-CoV-2 in IFN-γ-primed airway epithelial cell lines.

### GBP1 does not mediate antiviral activity through disruption of actin filaments

**Fig 6:**
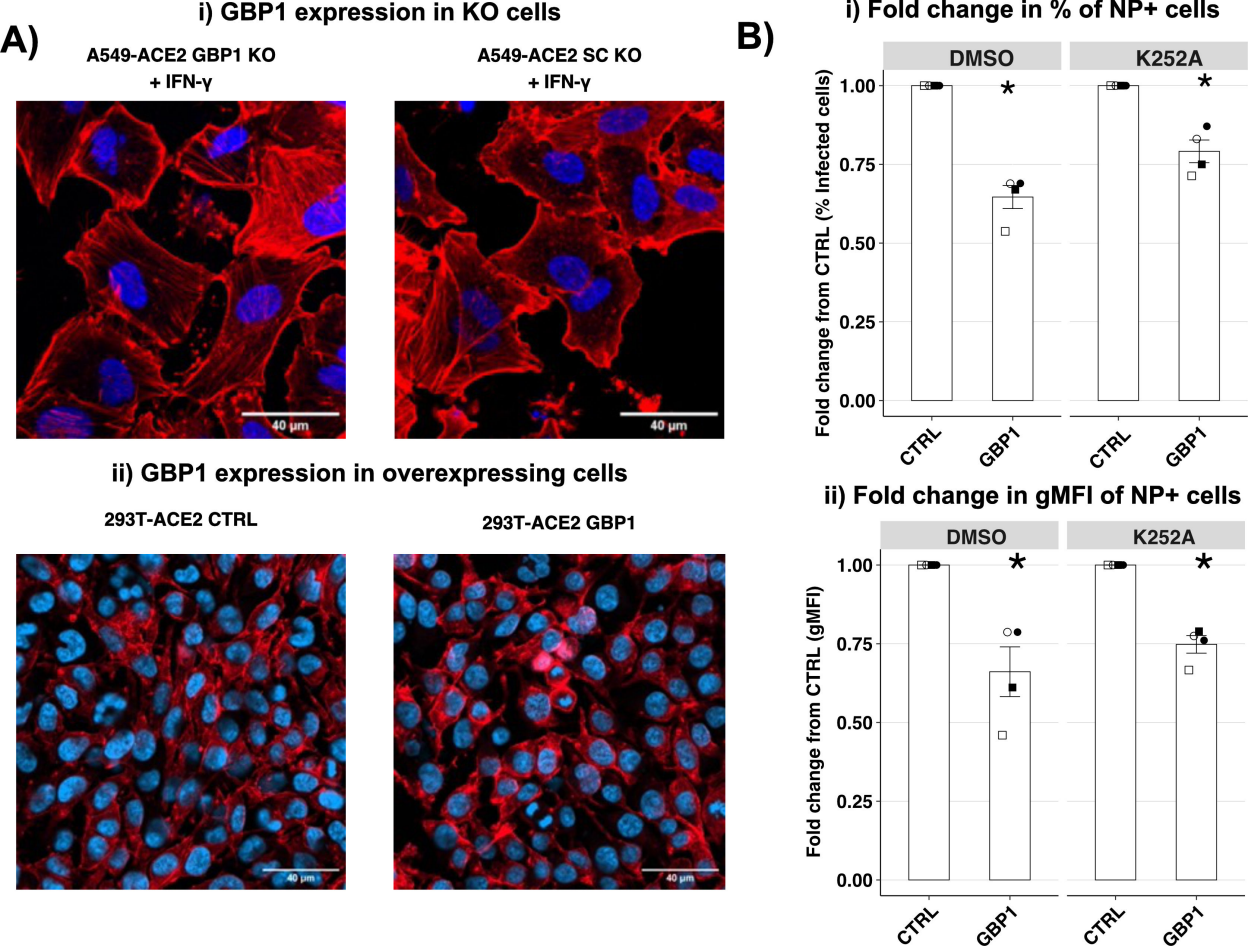
GBP1-mediated restriction of SARS-CoV-2 is not modulated by the actin pathway. (A) Confocal microscopy to assess the impact of endogenous or exogenous GBP1 expression on actin staining. (Ai) A549 ACE2-GBP1 KO or scrambled (–SC) control cells treated for 24 h with 100 ng/µL of IFN-γ to upregulate GBP1 expression were stained for expression of actin (red) and with DAPI to visualize cell nuclei (blue). (Aii) Stably transfected 293T-ACE2-CTRL and -GBP1 cells were stained to visualize both actin and cell nuclei. Images were acquired using LSM900 at 63× (A549-ACE2) or 20× (293T-ACE2) magnification. (B) 293T-ACE2-CTRL or -GBP1 cells were infected with Vic01 at MOI 0.5 and then treated 8 h later with 20 µM K252A or an equivalent volume of DMSO carrier. Cells were fixed and stained for intracellular expression of viral NP at 24 hpi and then analyzed by flow cytometry. The mean fold change of the percent of NP^+^ cells (top panel) and gMFI (bottom panel) relative to A549-293T-CTRL cells from four independent experiments (each with triplicate samples) is shown. Statistical analysis was performed using the Wilcoxon rank sum test to compare cell lines expressing GBP1 to the CTRL cell line. *P < 0.05, **P < 0.01, ***P < 0.001.

Previous studies have shown that overexpression of human GBP1 can disrupt the actin filaments and this, in turn, has been linked to its ability to inhibit the γ-herpesvirus virus, KSHV (22). To investigate if GBP1 disrupted actin filaments, we assessed the impact of IFN-γ-mediated upregulation of endogenous GBP1 in cells which do (A549-ACE2 scrambled [SC]) or do not (GBP1 KO) express endogenous GBP1 (Fig. 6A, upper panels). Using confocal microscopy, we observed no major differences in actin staining in IFN-γ-primed cells, which did or did not express endogenous GBP1. Similarly, no major differences in actin filament staining were observed in 293T-ACE2-GBP1 cells overexpressing exogenous GBP1 when compared to -CTRL cells (Fig. 6A, lower panels).

A previous study utilized high-resolution microscopy to show that the SARS-CoV-2 infection modified the filamentous (F) actin nanostructures in A549 cells, likely affecting viral assembly and egress (38). Moreover, this study showed that proteins, such as protein kinase N (PKN), which interacts with alpha-actinins to affect actin fiber rearrangements, were upregulated in cells following SARS-CoV-2 infection. It further used a PKN inhibitor to interfere with F-actin rearrangement, and this significantly reduced viral RNA levels. Given that GBP1 is also reported to interact with and remodel actin (39), we hypothesized that like PKN inhibitors, GBP1 upregulation might also interfere with F-actin rearrangement and therefore affect SARS-CoV-2 replication. Therefore, 293T-ACE-GBP1 and -CTRL cells were infected with SARS-CoV-2 and then treated with K252A (PKN inhibitor) or DMSO (carrier) at eight hpi, and the percentage of NP^+^ cells was determined at 24 hpi. Consistent with previous reports (38), the addition of K252A significantly reduced the percentage of NP^+^ SARS-CoV-2-infected 293T-ACE2-CTRL cells (Fig. S4). To determine the fold reduction across four independent experiments, results from 293T-ACE2-CTRL cells were assigned a value of 1.0, and the percentage or the gMFI of NP^+^ 293T-ACE2-GBP1 cells was determined relative to this. As seen in Fig. 6B, GBP1-mediated restriction of SARS-CoV-2 was still observed in K252A-treated cells (i.e., both the percentage and the gMFI of NP^+^ -GBP1 cells were significantly reduced compared to -CTRL cells). These studies indicate that GBP1-mediated remodeling of actin does not appear to be the predominant mechanism by which GBP1 restricts SARS-CoV-2 replication, at least in the cell types tested in our studies.

### GBP1 does not inhibit the infectivity of pseudotyped viruses expressing the spike protein of SARS-CoV-2

**Fig 7:**
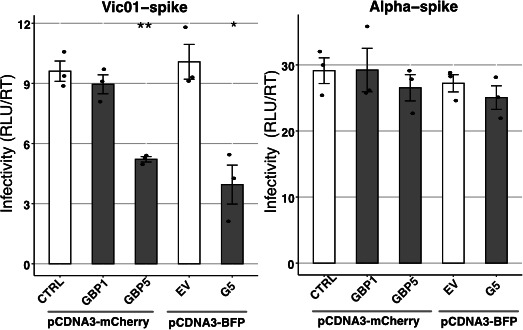
GBP1 inhibits SARS-CoV-2 by a mechanism that is distinct to that reported for GBP5. Pseudoviruses (PVs) expressing SARS-CoV-2 Wuhan or Alpha spike proteins were produced in 293T/17 cells transfected with pcDNA3.1-mCherry vectors to express CTRL, GBP1, or GBP5 or with control pcDNA3.1 BFP vectors that were empty (EV) or expressing GBP5. PV infectivity was measured by titration and luciferase assay on Caco2 cells and normalized to reverse transcriptase activity of input virus measured by qPCR. Data show triplicate samples from one of two independent experiments performed with similar results. Statistical analysis was performed using Student’s unpaired *t*-test with unequal variance to compare cell lines expressing ISG to the CTRL cell line. *P < 0.05, **P < 0.01, ***P < 0.001.

Human GBP2 and GBP5 can inhibit furin-mediated processing of viral glycoproteins expressed by a number of different RNA viruses (34), including recent studies confirming their ability to inhibit processing of the SARS-CoV-2 spike glycoprotein (20). Less is known regarding the ability of GBP1 to inhibit furin-mediated processing, although interactions between swine GBP1 and furin were correlated with reduced infectivity of Japanese encephalitis virus (JeV) (40). Given that Mesner et al. demonstrated that overexpression of human GBP2 or GBP5 reduced the infectivity of PVs expressing the SARS-CoV-2 spike protein (20), we used similar approaches to determine the impact of GBP1 on furin-mediated processing of the SARS-CoV-2 spike. An advantage of the PV system is that it allows the impact of particular GBP proteins on the spike to be assessed independently of other SARS-CoV-2 viral proteins. Therefore, PVs expressing SARS-CoV-2 spike protein (Vic01 or Alpha VOC) were generated in cells transfected with GBP1 or GBP5 or CTRL pcDNA3.1-mCherry plasmids or in cells transfected with GBP5 pcDNA3.1 BFP plasmid or with empty vector (EV) control, as previously reported (20). To focus on the ability of GBPs to inhibit furin-mediated cleavage of spike, PVs were titrated on Caco2 cells which express both ACE2 and TMPRSS2 and require furin pre-cleavage of spike to promote efficient SARS-CoV-2 entry (41, 42). For PV expressing the Vic01 spike, expression of GBP5 in PV-producing 293T cells significantly reduced particle infectivity, whereas GBP1 did not (Fig. 7). Consistent with previous findings (20), PVs expressing spike protein from the Alpha VOC were not sensitive to GBP5-mediated restriction, nor did GBP1 expression affect their infectivity. Thus, while GBP5 inhibits furin-mediated cleavage of Vic01, but not the Alpha VOC spike protein, GBP1 does not inhibit either. This indicates that the antiviral activity of GBP1 against SARS-CoV-2 is clearly distinct and is not mediated by inhibition of furin-mediated cleavage of the viral spike protein.

### The chromosome 3 cluster of mouse GBPs does not contribute to inhibition of SARS-CoV-2 replication *in vivo*

**Fig 8:**
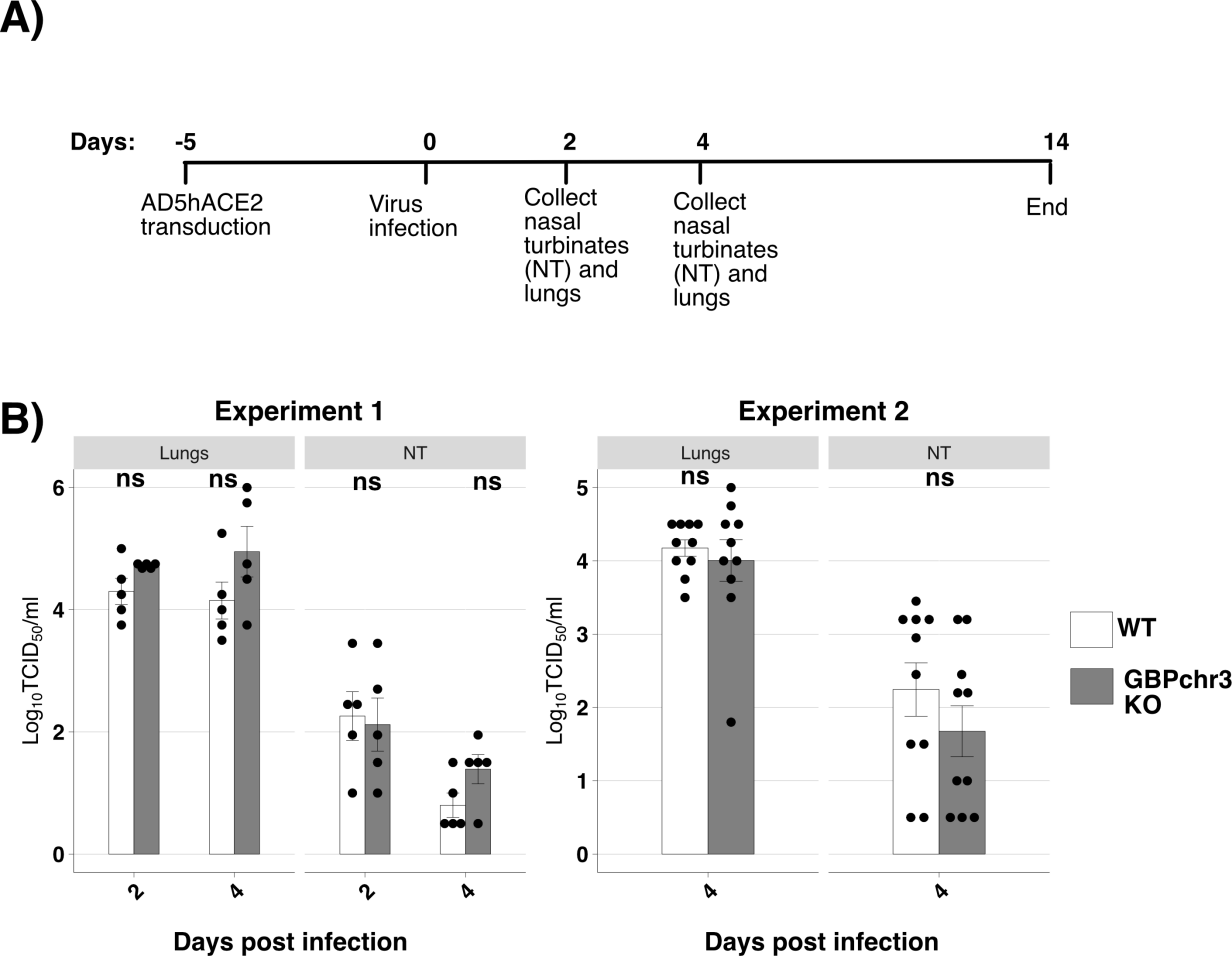
The presence or absence of the chromosome 3 cluster of mouse GBPs does not alter SARS-CoV-2 replication in a mouse model of infection. (A) Schematic and timeline of experimental setup for mouse experiments. Briefly, mice were transduced with adenovirus vector (2.5 × 10^8^ FFU) to express hACE2, 5 days prior to intranasal inoculation with SARS-CoV-2 infection. Organs were harvested at indicated time points and virus titers determined. (B) Wild-type (WT) or knockout mice lacking the chromosome 3 cluster of mouse GBPs (mGBPchr3 KO) were infected via the intranasal route with 50 µL of PBS containing 10^4^ TCID_50_ of SARS-CoV-2 (Vic01). At 2 or 4 days post-infection (dpi), mice were euthanized, and virus titers in homogenates prepared from nasal tissues and lungs were determined by TCID_50_ assay on Vero cells. Titers from experiment 1 (*n* = 5 mice/group) and experiment 2 (*n* = 10 mice/group) are shown. Student’s unpaired *t*-test with unequal variance was used to compare viral titers in WT mice to titers from GBPchr3 KO mice. *P < 0.05, **P < 0.01, ***P < 0.001.

Unlike humans, mice have 11 GBPs and two GBP pseudogenes, distributed across two chromosomes clusters. Of the mouse GBPs, *mGBP1*, *mGBP2*, *mGBP3*, *mGBP5*, *mGBP7,* and one pseudogene are on chromosome 3, whereas *mGBP4*, *mGBP6*, *mGBP8*, *mGBP9*, *mGBP10*, *mGBP11*, and the second pseudogene are on chromosome 5 (43). Phylogenetic organization of human and mouse GBPs shows that they form separate clusters, with mGBP1 and mGBP2 being the closest to human GBP1 (44). Given that human GBP1 and GBP2/5 (20) mediate antiviral against SARS-CoV-2 *in vitro*, it was of interest to determine if replication of SARS-CoV-2 would be affected in mice lacking the chromosome 3 cluster of mouse GBPs (mGBPchr3 KO mice) (30). In these studies, wild-type (WT) and GBPchr3 KO mice were transduced with an adenovirus vector to express hACE2 in the airways 5 days prior to challenge with SARS-CoV-2 (Vic01, 10^4^ TCID_50_/mouse) and then monitored and weighed daily for up to 14 days (Fig. 8A). Both WT and mGBP3ch3 KO mice showed negligible weight loss over the first 5 days of infection, and weight gain was observed in both groups after this time (Fig. S5). To assess virus replication, mice (*n* = 5 mice/group) were euthanized at 2 or 4 days post-infection (dpi), and titers of infectious virus in clarified homogenates prepared from lung or nasal turbinates were determined by TCID_50_ on Vero cells. No significant differences were observed in virus titers recovered from WT or mGBPchr3 KO mice, though noting there was a modest enhancement of virus titers in the lungs of mGBPchr3 KO mice at 4 dpi (Fig. 8B, left panel), although this was not significant. An additional experiment focused on 4 dpi (*n* = 10 mice/group) confirmed no significant differences in the virus titers recovered from WT versus mGBPchr3 KO mice (Fig. 8B, right panel). Thus, while multiple human GBPs can inhibit SARS-CoV-2 replication *in vitro*, mouse GBPs from the chromosome 3 cluster do not impact virus replication in a mouse model of SARS-CoV-2 infection.

## DISCUSSION

Human GBP1, an ISG protein with GTPase activity, is readily induced by Type I and II interferons (45) and has been reported to mediate antiviral activity against RNA viruses including influenza A virus (IAV), vesicular stomatitis virus (VSV), encephalomyocarditis virus (EMCV), hepatitis E virus (HEV), hepatitis C virus (HCV) (35, 46–48), and DNA viruses, such as KSHV (22). Herein, using overexpression of exogenous GBP1, as well as knockdown/knockout of endogenous GBP1, we demonstrated the antiviral activity of human GBP1 against replication-competent SARS-CoV-2 isolates, including Vic01, as well as VOCs Alpha, Beta, Delta, Omicron BA.1, and Omicron BA.2. Similar to other viruses, such as IAV and KSHV, we show that restriction of SARS-CoV-2 is dependent on the GTPase activity of GBP1 (22, 35), which is distinct from other GBPs such as GBP5, where restriction of HIV-1 is independent of GTPase activity (49). Notably, we find that the antiviral activity of GBP1 is not mediated through targeting spike maturation like GBP2/5 (20, 34), or modulation of the actin pathway. Instead, we show GBP1-mediated inhibition of SARS-CoV-2 occurs via a distinct mechanism that impacts viral protein levels.

While GBP1 has been reported to restrict several viruses, the mechanisms by which it mediates antiviral activity often remain undefined. For human GBP1, it is known that a functional GTPase domain is required for inhibition of IAV and HCV (35, 48), but further mechanistic details are lacking. Human GBP1 has also been shown to inhibit VSV and ECMV, but again, the mechanisms of action were not investigated or defined (46). GBP1-mediated inhibition of HEV occurred independently of its GTPase activity but demonstrated that GBP1 dimerization was required to localize HEV viral capsids to the lysosomal pathway for subsequent degradation (47). Of interest, while SARS-CoV-2 also utilizes the lysosomal pathway, in this case, it is to promote viral egress (50, 51). Therefore, if GBP1 were to localize SARS-CoV-2 proteins to the lysosome, it would be expected to promote virus replication rather than mediate the antiviral effects observed in our studies. Previous studies investigating chicken GBP1 demonstrated its ability to inhibit infectious bronchitis virus (IBV), a virus from the gamma coronavirus family, by degrading the viral N protein through the autophagy pathway (52). It would be interesting in future studies to test if a similar mechanism of action applies to human GBP1 and SARS-CoV-2, though it is worth noting that the sequence similarity between human and chicken GBP1 is quite low at 44%.

In other studies investigating GBP1-mediated virus restriction, overexpression of human GBP1 led to disruption of actin filaments, which, in turn, was linked to inhibiting nuclear delivery of KSHV virions during the early stages of virus replication, which ultimately led to a reduction in viral titers (22). Porcine GBP1 has approximately 80% similarity in sequence to human GBP1 (53), and inhibition of porcine reproductive respiratory syndrome virus (PRRSV) and pseudorabies virus (PRV) by porcine GBP1 has also been linked to disruption of actin filaments (54, 55). Like KSHV, disruption of actin filaments by porcine GBP1 also reduced nuclear import of PRV virions (54). While both SARS-CoV-2 and PRRSV are positive-strand RNA viruses, restriction of these viruses by human and porcine GBP1, respectively, appears to occur via distinct mechanisms. For example, restriction of PRRSV by porcine GBP1 was associated with inhibition of viral mRNA at 24 hpi, and we report that human GBP1 did not impact SARS-CoV-2 transcription or replication (Fig. 4). Moreover, the mechanism by which disruption of actin filaments contributed to PRRSV restriction was not determined (55).

Herein, GBP1 overexpression in human epithelial cells (A549-ACE2 and 293T-ACE2) did not result in overt disruption of actin filaments, which may indicate cell type-specific effects, as other studies utilized SLK renal carcinoma endothelial cells (22), PK-15 porcine kidney cells (54), or MARC-145 African green monkey kidney cells (55). We also utilized a PKN inhibitor to determine if human GBP1 modulated SARS-CoV-2 replication via interactions with actin. Swain et al. utilized various approaches to demonstrate that rearrangement of F-actin was associated with the late phases of productive SARS-CoV-2 infection in airway epithelial cells (38). Specifically, they used PKN inhibitor K252A to prevent PKN-induced reorganization of actin filaments (56), which, in turn, inhibited SARS-CoV-2 replication. However, in our studies, human GBP-1-mediated restriction of SARS-CoV-2 was evident in addition to the reduced levels of SARS-CoV-2 replication observed in cells treated with K252A (Fig. 6B). Together, these findings suggest that human GBP1-mediated restriction of SARS-CoV-2 can occur independently of the PKN-actin axis.

We also investigated whether human GBP1 affected SARS-CoV-2 replication by modulating furin-mediated cleavage of the viral spike protein. Previously, porcine GBP1 was reported to inhibit furin-mediated cleavage of viral pRM proteins to restrict JeV replication (40). Human GBP2 and GBP5 have also been reported to restrict a number of RNA viruses, including SARS-CoV-2, by inhibiting furin-mediated cleavage of viral envelope glycoproteins (20, 34). However, in contrast to GBP5, overexpression of human GBP1 did not affect the infectivity of PVs expressing the SARS-CoV-2 spike (Fig. 7), confirming that human GBP1 does not inhibit processing of SARS-CoV-2 spike.

In addition to human GBP1, we explored the role of mouse (m)GBPs utilizing mGBPchm3 KO mice that do not express multiple mGBPs (mGBP1/2/3/5/7). These mice have been used to demonstrate the importance of mGBPs in many studies, including in IFN-γ-mediated immunity against *Toxoplasma gondii* (30) and in inflammasome activation in macrophages following infection with *Francisella novicida* (57). We observed no differences in SARS-CoV-2 titers from the lungs or nasal tissues of WT mice vs. GBPchm3 KO mice. These observations are consistent with our recent findings that mouse GBPs from chromosome cluster 3 did not modulate replication of diverse viruses (IAV, HSV-1, SeV, LCMV, or EMCV) *in vitro*, nor did we observe differences in viral titers following infection of GBPchm3 KO mice with IAV, HSV-1, or LCMV (58). Of note, our previous study also confirmed that mGBP1/2/3/5/7 could be induced by IFNs in a mouse lung airway epithelial cell line (LA-4 cells) or in lung fibroblasts from WT mice (58). We have also utilized overexpression approaches and mGBP1 KO mice to demonstrate that mGBP1 does not inhibit IAV replication *in vitro* or *in vivo* (59), despite previous reports of human GBP1 mediating potent antiviral activity against IAV *in vitro* (35). Thus, our findings with SARS-CoV-2 add to a growing body of evidence that mouse and human GBPs may be functionally distinct with regard to antiviral activity.

In summary, this study demonstrates for the first time that human GBP1 can restrict SARS-CoV-2 replication *in vitro*. Our data would indicate that neither processing of the spike protein nor GBP1-mediated modulation of actin filaments contributes markedly to the ability of GBP1 to inhibit SARS-CoV-2 by reducing protein levels. Future studies could investigate if GBP1-mediated inflammasome activation might contribute to its antiviral activity, given that this is well established in the context of GBP1-mediated antibacterial immunity (60–62). Results from SARS-CoV-2 infection of mGBPchr3 KO mice also add further support to the notion that GBPs differ in their ability to contribute to antiviral defense in humans and in mice. While a more detailed mechanistic understanding of GBP1-mediated inhibition of SARS-CoV-2 is needed, this study highlights the potential of GBP1 and/or other GBPs as potential targets for the future development of host-directed antiviral therapies. It also provides insights regarding the limitations of mouse models to gain insight regarding the *in vivo* activity of GBP-family proteins, particularly when *in vitro* activity has been defined for specific human GBPs.

## Data Availability

The data that support the findings of this study are available in Materials and Methods and the supplemental material.

## SUPPLEMENTAL MATERIAL

Supplemental figures jvi.00823-25-s0001.docx Fig. S1 to S5.

Table S1 jvi.00823-25-s0002.xlsx Codon-optimized sequence of restriction factors and ACE2 used for overexpression.

ASM does not own the copyrights to Supplemental Material that may be linked to, or accessed through, an article. The authors have granted ASM a non-exclusive, world-wide license to publish the Supplemental Material files. Please contact the corresponding author directly for reuse.
