# Supplementary material for: Human guanylate-binding protein (GBP) 1 inhibits replication of severe acute respiratory syndrome coronavirus 2
Source: J Virol. 2025 Sep 15;99(10):e00823-25. doi: 10.1128/jvi.00823-25 (PMC12548390; doi:10.1128/jvi.00823-25)
Supplement: Supplemental figures — Fig. S1 to S5. [file jvi.00823-25-s0001.docx]

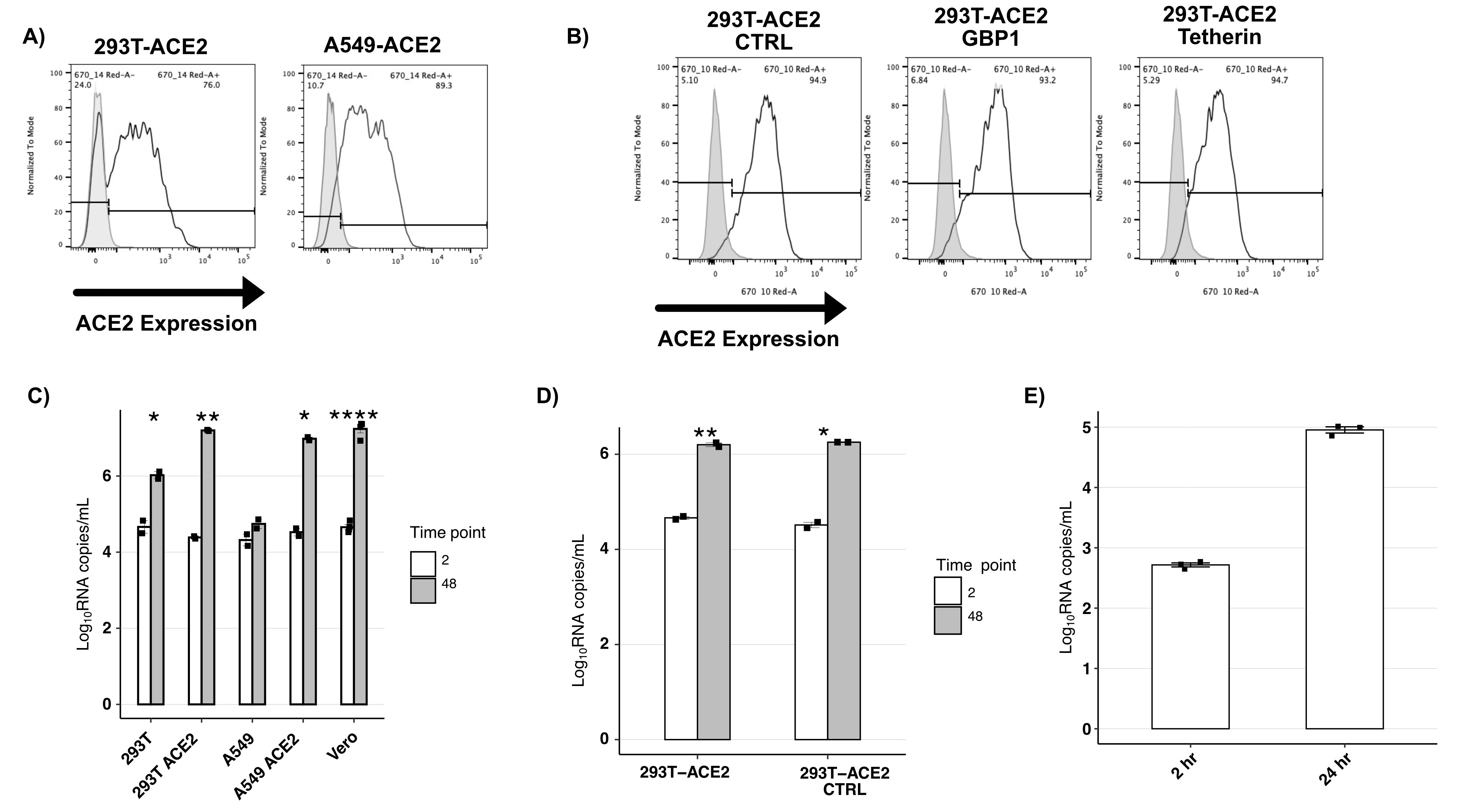


**Figure S1: Generation of human cell lines with stable overexpression of cell-surface ACE2. A)** Following retroviral transduction, A549 and 293T cells were stained for cell surface expression of ACE2 and examined by flow cytometry. Parental (grey histograms) and stably transduced cells (white histograms) are shown. **B)** 293T-ACE2 cells were transfected to express ISG proteins of interest using pcDNA3.1 vectors. The ACE2 surface expression of cells overexpressing CTRL, GBP1 or Tetherin was also examined using flow cytometry. Parental (grey histogram) and stably transfected cells (white histograms) are shown**.**  **C)** Parental 293T, 293T-ACE2, parental A549, A549-ACE2 and Vero cell controls were infected with SARS-CoV-2 (MOI 0.1) and virus titres in clarified supernatants were determined by qPCR at 2 and 48 hours post infection (hpi). **D)** 293T-ACE2 cells and 293T-ACE2 cells stably overexpressing an irrelevant control protein (293T-ACE2 CTRL) were infected with SARS-CoV-2 (Vic 01, MOI 0.1), and virus titres in clarified supernatants were determined by qPCR at 2 and 48 hpi. Data show triplicate samples from one of two independent experiments performed with similar results. **E)** 293T-ACE CTRL cells were infected with Vic 01 (MOI 0.1) for 2 or 24 hrs, total cellular RNA was extracted and then levels of genomic+sg RNA was measured by qPCR. Statistical analysis was performed using Student’s unpaired t-test with unequal variance compare cell lines expressing ISG to the CTRL cell line. * p<0.05, ** p<0.01, *** p<0.001


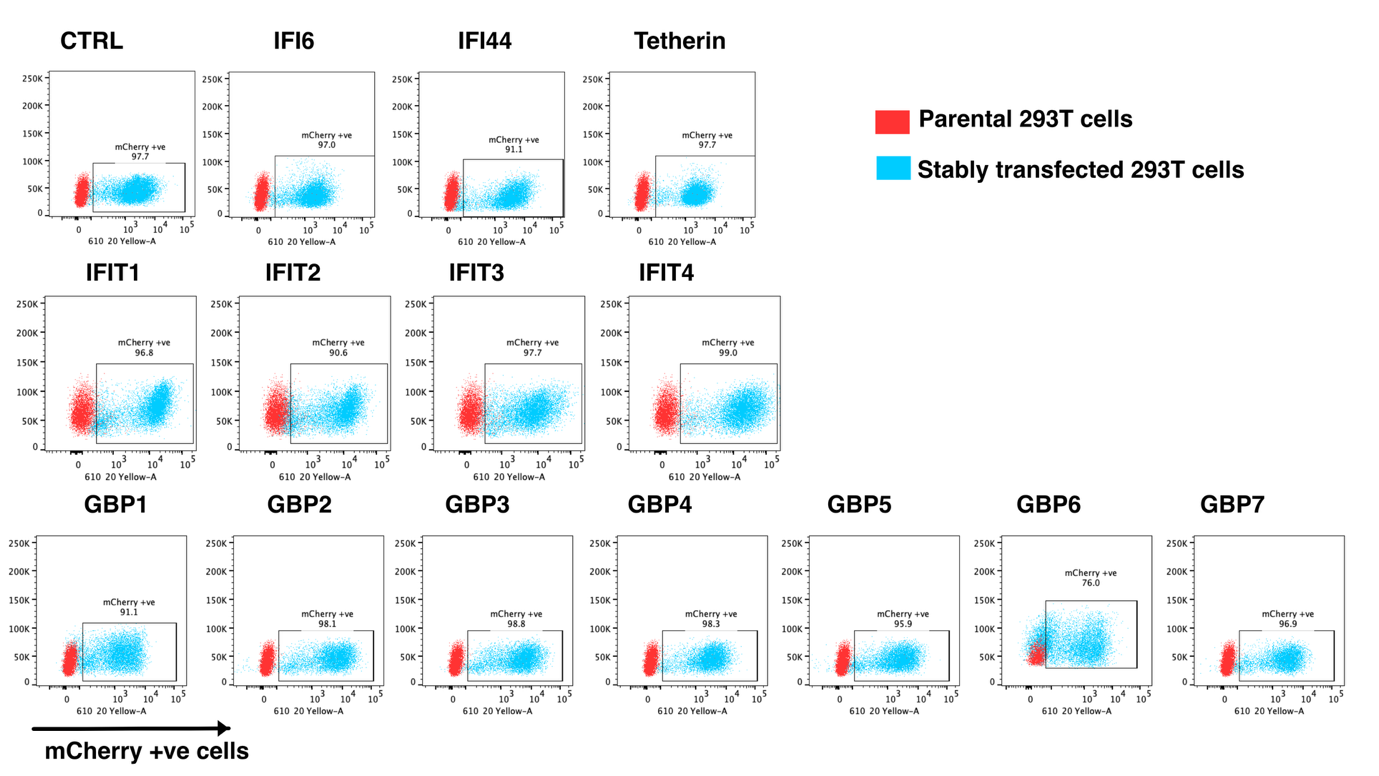


**Figure S2: Generation of 293T-ACE2 cells with stable overexpression of different ISG proteins.** 293T-ACE2 cells were transfected with pcDNA3.1-mCherry vectors expressing ISG proteins of interest, each with a N-terminal FLAG tag, or with the same vector expressing cytoplasmic chicken ovalbumin with no FLAG tag as a control (CTRL). Stable transfectants were selected in the presence of hygromycin and enriched by sorting for mCherry^+^ cells. The percentage of mCherry^+^ cells following selection was measured by flow cytometry.


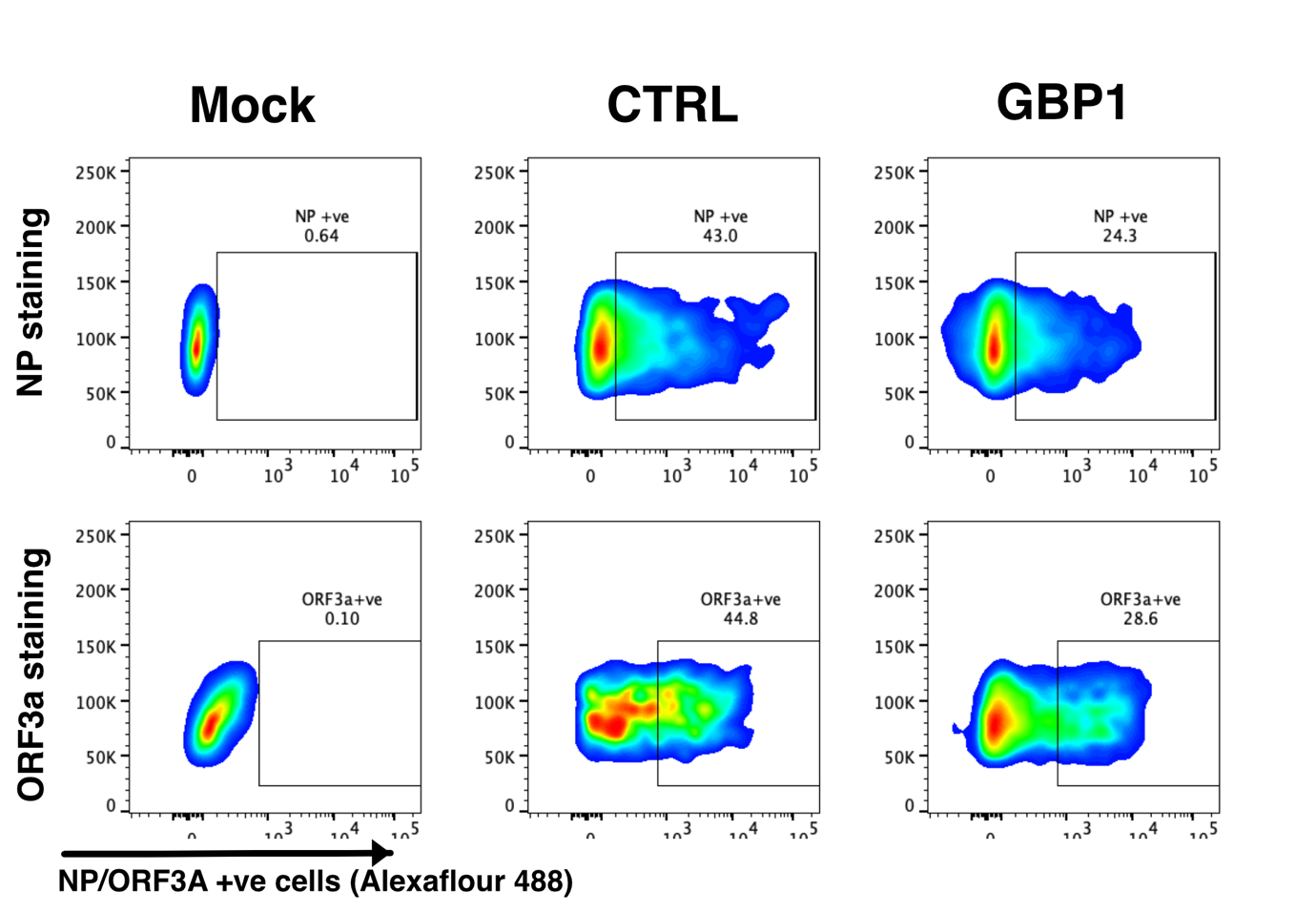


**Figure S3: SARS-CoV-2-infected cells overexpressing GBP1 show a reduced percentage of viral NP and ORF3a proteins, as well as a reduced gMFI.** 293T-ACE2-CTRL or 293T-ACE2 GBP1 cells were mock-infected or infected with SARS-CoV-2 (Vic01, MOI 0.1) for 1 hr, washed and then cultured at 37^o^C. At 24 hpi, cells were fixed, stained for intracellular expression of either viral NP or ORF3a and analysed by flow cytometry. Representative FACs plots for NP and ORF3a staining cells are shown from Mock, and for SARS-CoV-2-infected CTRL or GBP1 overexpressing cells are shown.


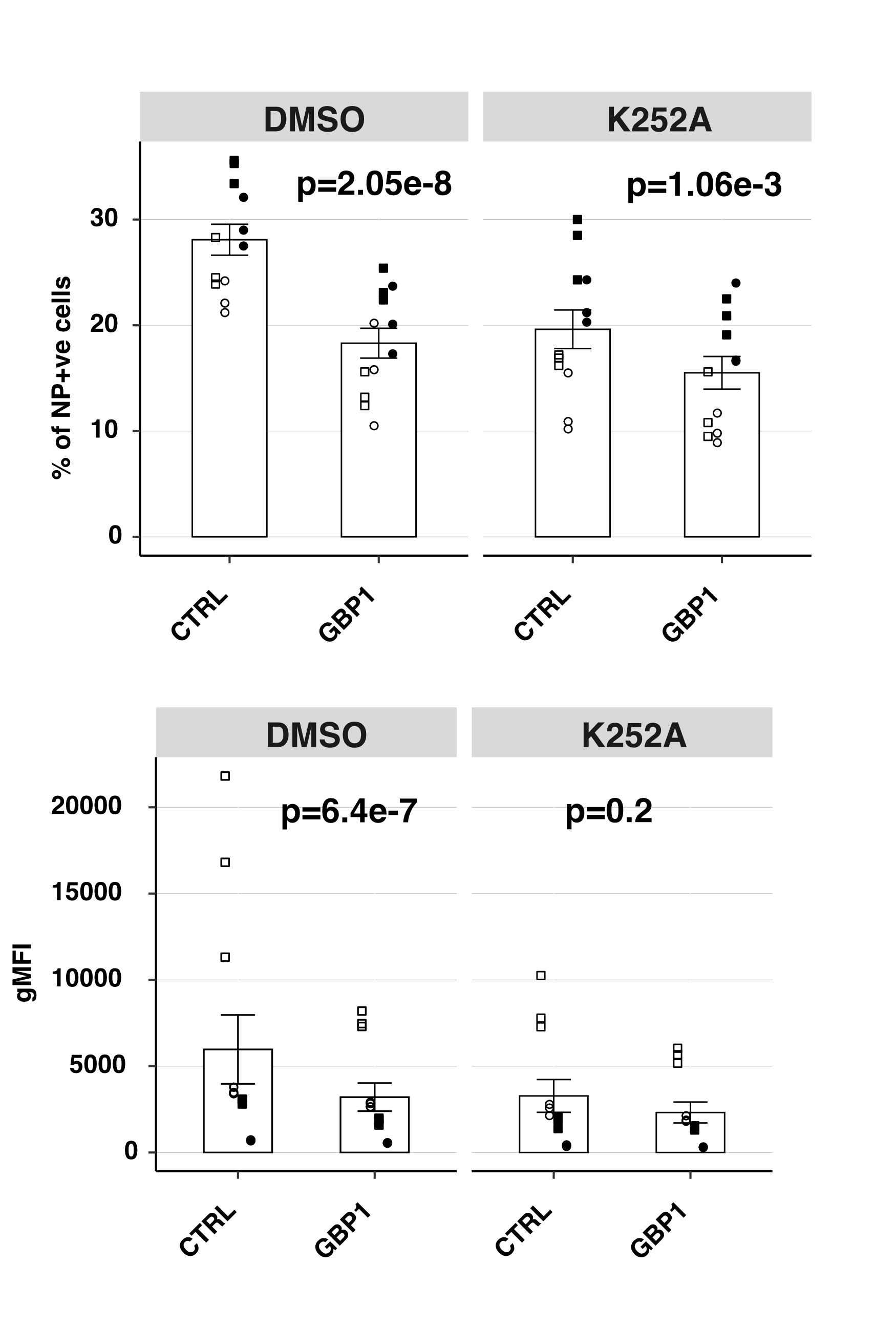


**Figure S4:** 293T-ACE2-CTRL or -GBP1 cells were infected with Vic01 at MOI 0.5 and then treated with 20uM K252A or equivalent volume of DMSO 8 hpi. Viral infection was determined at 24 hpi when cells were fixed, stained for intracellular expression of viral NP and analysed by flow cytometry. Raw data pooled from four independent experiments are shown (a different symbol is used for each biological replicate). Statistical analysis was performed using a mixed effects model to compare differences between CTRL and GBP1-overexpressing cells utilising data points from all experiments as described in Materials and Methods. The *p* value for each CTRL vs GBP1 comparison under each treatment condition is indicated on figure.


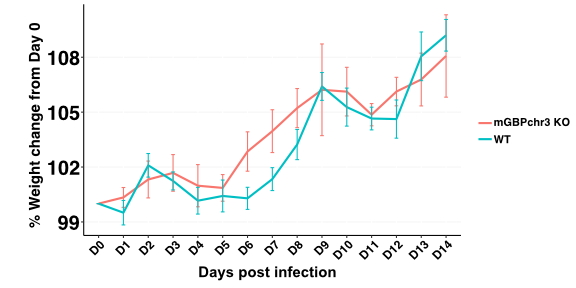


**Figure S5:** Wild type (WT) or knockout mice lacking the chromosome 3 cluster of mouse GBPs (mGBPchr3 KO) were infected via the intranasal route (n = 5/group) with 50 ul of PBS containing 10^4^ TCID_50_ of SARS-CoV-2 (Vic01) on day 0. Mice were monitored and weighed daily for 14 days. Results show percentage change in weight from day 0 (mean± SEM).
